# Analgesic Modalities in Patients Undergoing Open Pancreatoduodenectomy—A Systematic Review and Meta-Analysis

**DOI:** 10.3390/jcm12144682

**Published:** 2023-07-14

**Authors:** Simona Mărgărit, Adrian Bartoș, Laura Laza, Cristiana Osoian, Robert Turac, Oszkar Bondar, Daniel-Corneliu Leucuța, Lidia Munteanu, Horațiu Nicolae Vasian

**Affiliations:** 1 Department of Anesthesia and Intensive Care, “Iuliu Hațieganu” University of Medicine and Pharmacy, 400012 Cluj-Napoca, Romania; simona.margarit@umfcluj.ro (S.M.); horatiu_vasian@umfcluj.ro (H.N.V.); 2 “Prof. Dr. Octavian Fodor” Regional Institute of Gastroenterology and Hepatology, 400162 Cluj-Napoca, Romaniaciobanulidia@yahoo.com (L.M.); 3 Department of Surgery, “Iuliu Hațieganu” University of Medicine and Pharmacy, 400012 Cluj-Napoca, Romania; 4 Department of Medical Informatics and Biostatistics, “Iuliu Hațieganu” University of Medicine and Pharmacy, 400012 Cluj-Napoca, Romania; dleucuta@umfcluj.ro; 5 Department of Internal Medicine, “Iuliu Hațieganu” University of Medicine and Pharmacy, 400012 Cluj-Napoca, Romania

**Keywords:** open pancreatoduodenectomy, postoperative pain, opioids, neuraxial, wound infiltration, truncal block

## Abstract

Background: This systematic review explored the efficacy of different analgesic modalities and the impact on perioperative outcome in patients undergoing pancreatoduodenectomy. Methods: A systematic literature search was performed on PubMed, Embase, Web of Science, Scopus, and Cochrane Library Database using the PRISMA framework. The primary outcome was pain scores on postoperative day one (POD1) and postoperative day two (POD2). The secondary outcomes included length of hospital stay (LOS) and specific procedure-related complications. Results: Five randomized controlled trials and ten retrospective cohort studies were included in the systematic review. Studies compared epidural analgesia (EA), patient-controlled analgesia (PCA), continuous wound infiltration (CWI), continuous bilateral thoracic paravertebral infusion (CTPVI), intrathecal morphine (ITM), and sublingual sufentanil. The pain scores on POD1 (*p* < 0.001) and POD2 (*p* = 0.05) were higher in the PCA group compared with the EA group. Pain scores were comparable between EA and CWI plus PCA or CTPVI on POD1 and POD2. Pain scores were comparable between EA and ITM on POD1. The procedure-related complications and length of hospital stay were not significantly different according to the type of analgesia. Conclusions: EA provided lower pain scores compared with PCA on the first postoperative day after pancreatoduodenectomy; the length of hospital stay and procedure-related complications were similar between EA and PCA. CWI and CTPVI provided similar pain relief to EA.

## 1. Introduction

The postoperative pain after open pancreatoduodenectomy (PD) is significant owing to big incisions and extensive abdominal dissection associated in many cases with preoperative pain and opioid use prior to surgery [1,2]. Inadequate pain control affects the neuroendocrine stress response, increases complication rates, and causes delayed functional recovery and prolonged hospital stays [3,4,5]. Pain management is a key component of enhanced recovery after surgery (ERAS) protocol after PD [6], but optimal pain control continues to be a challenge.

Epidural analgesia (EA) using either continuous epidural or patient-controlled administration and intravenous opioid analgesia (most frequently used as patient-controlled analgesia (PCA)) are still the most popular techniques for analgesia following pancreatoduodenectomy. Thoracic EA provided better pain control with less perioperative opioid consumption [1,5,7,8,9,10] and a faster return of a postoperative intestinal function [11,12,13]. However, despite good analgesia, thoracic epidural analgesia carries risks of technique-specific complications such as spinal hematoma [7,14] as well as a high failure rate of treatment (between 28% and 50%) [15,16,17,18,19], primarily because of inadequate pain relief or hemodynamic instability. Excessive fluid administration and vasopressors associated with EA contribute to postoperative morbidity, including impairment of enteric anastomosis [16,19,20,21,22]. The clinical benefit of EA in terms of reduced morbidity and mortality is not so conclusive [12,23,24,25,26,27].

On the contrary, intravenous PCA, one of the most common strategies for pain relief following pancreatoduodenectomy, offers effective pain relief, but with higher opioid consumption. Furthermore, it does not promote hypotension; consequently, the need for vasopressors and fluids is lower than EA [5,12,27,28]. However, their related side effects such as respiratory depression, ileus, nausea, vomiting, and long-term opioid dependence after surgery [2,5,29], as well as the evidence of a link between opioids and the growth of cancer cells [2,30], have raised some concerns about their use. Thus, some strategies of sparing opioids other than EA have become more common in major abdominal surgery.

Continuous infusion of local anesthetic par transabdominal wound catheter or wall catheter (transversus abdominis plane block) associated with PCA provides effective pain relief in major abdominal surgery, reduced opioid consumption, and the risk of complications such as hypotension and block failure compared with EA [31,32]. The bilateral paravertebral block provides better pain relief and reduced opioid consumption in the early postoperative period of abdominal surgery [33]. However, few data are available on pancreatic surgery [33,34,35,36,37].

Single-shot intrathecal morphine (ITM) is an effective strategy for postoperative analgesia following abdominal surgery, having a prolonged duration of action and higher potency [38]. ITM reduces pain at rest and on movement on the first postoperative day with low complication risk [39]. ITM, as a part of a multimodal pain management strategy in major hepatopancreatic biliary surgery, provides effective pain relief, requires less fluid therapy, and reduces the length of hospital stay compared with EA [40,41,42]. However, sparse data are available on pancreatoduodenectomy [40,43,44].

Sublingual sufentanil tablets (SSTs) as an alternative to EA seem to be an attractive analgesic option for pain management in major abdominal surgery and pancreatic surgery [45,46].

The present systematic review and meta-analysis aimed to summarize and compare the efficacy and safety of different analgesic modalities in patients undergoing pancreatoduodenectomy.

## 2. Material and Methods

This systematic review and meta-analysis were reported in accordance with the recommendations from the Preferred Reporting Items for Systematic Reviews and Meta-Analyses (PRISMA) guidelines [47]. No prior registration of study protocol was undertaken for this review.

### 2.1. Eligibility Criteria

The inclusion criteria were randomized controlled trials (RCTs) or observational cohort studies published in English language that compare two or more analgesic techniques and reported at least one outcome of interest.

Studies that included minimally invasive cases (laparoscopic or robotic) or those that included open pancreatic resection (pancreatoduodenectomy and distal pancreatectomy) were excluded unless subgroup analysis of pancreatoduodenectomy was available. Case reports, conference abstracts, previously reported systematic reviews, studies with no full text available, and studies with fewer than ten patients were excluded too.

### 2.2. Information Sources and Search Strategy

We performed a systematic search of the electronic databases PubMed, Embase, Web of Science, Scopus, and Cochrane Library Database from 1 January 1995 through 1 October 2022. The following search terms were used (“pancreaticoduodenectomy” OR “duodenopancreatectomy OR “pancreas surgery” OR “pancreatic resection” OR “pancreatectomy” OR “Whipple operation/resection“ OR “pancreatoduodenectomy” OR “pancreatoduodenal resection“) AND (“analgesia” OR “pain control” OR “pain management” OR “postoperative pain” OR “opioid analgesia” OR “narcotic” OR” regional analgesia” OR “epidural analgesia” OR “neuraxial analgesia” OR “spinal block’ OR ‘intrathecal block “ OR “patient-controlled analgesia” OR “wound catheter” OR “TAP block” OR “paravertebral block”). The complete literature search is provided in Appendix S1 (Supplementary Information).

### 2.3. Selection Process

Two independent reviewers (L.L., O.C.) screened the titles and abstracts of all articles for relevance. Eligible full-text articles were subsequently reviewed by two other independent reviewers (R.T., O.B.). In case of any disagreement between the two reviewers, a third reviewer (L.M.) helped make the selection. Manual screening of the reference lists in relevant articles was conducted to identify any additional articles.

### 2.4. Data Collection Process and Items

Data were extracted by two independent reviewers directly from articles (L.L., R.T.). The following study characteristics were collected: country, study type, recruitment dates, centers, type of pancreatic resection, and analgesic modalities.

### 2.5. Primary and Secondary Outcomes Measurements

The primary outcome measures were pain scores on postoperative day 1 (POD1) and day 2 (POD2). Pain scores were rated on the numerical rating scale (NRS) from 0 to 10, where 0 indicated no pain and 10 correlated to the worst pain possible. In articles that used VAS, these were converted to the corresponding number on the NRS [48].

The secondary outcome measures included length of hospital day (LOS) and specific complications: postoperative pancreatic fistula, bile leakage, delayed gastric emptying (DGE), postoperative ileus, and gastrointestinal bleeding.

### 2.6. Study Quality Assessment

All studies included in the final analysis were assessed by two reviewers (M.S., D.C.L.) The risk of bias was evaluated using the Cochrane Risk-of-Bias tool 2.0 [49] for RCTs and the ROBINS-I tool [50] for non-randomized studies.

### 2.7. Statistical Analysis

The meta-analysis was performed using R environment for statistical computing and graphics (R Foundation for Statistical Computing, Vienna, Austria), version 4.1.2 [51], and the meta-R package [52].

For qualitative outcomes, the number of events and the total number of subjects in both intervention groups were extracted. For quantitative outcomes, the mean and SD were extracted. Data presented in the graphs of articles were extracted by Web plot digitizer [53]. Several equations were used prior to analysis for the estimation of mean and SD from several studies that reported the median, range, interquartile range, and sample size [54]. We calculated the random effects estimates for all outcomes because we presumed the presence of clinical variability between studies. To measure the heterogeneity variance for continuous outcomes, we used limited maximum likelihood and computed the mean difference as effect size. For qualitative outcomes, we used the Mantel–Haenszel estimator and computed odds ratios as effect size. For all estimators, a 95% confidence interval was calculated too. Heterogeneity was assessed using the I2 statistic; a threshold of 50% suggested moderate heterogeneity and 75% indicated substantial heterogeneity [55]. In the case of significant heterogeneity, sensitivity analyses were used using the leave-one-out method. A *p*-value of <0.05 was considered significant. The possible publication bias risk was assessed by the Egger test.

## 3. Results

**Figure 1:**
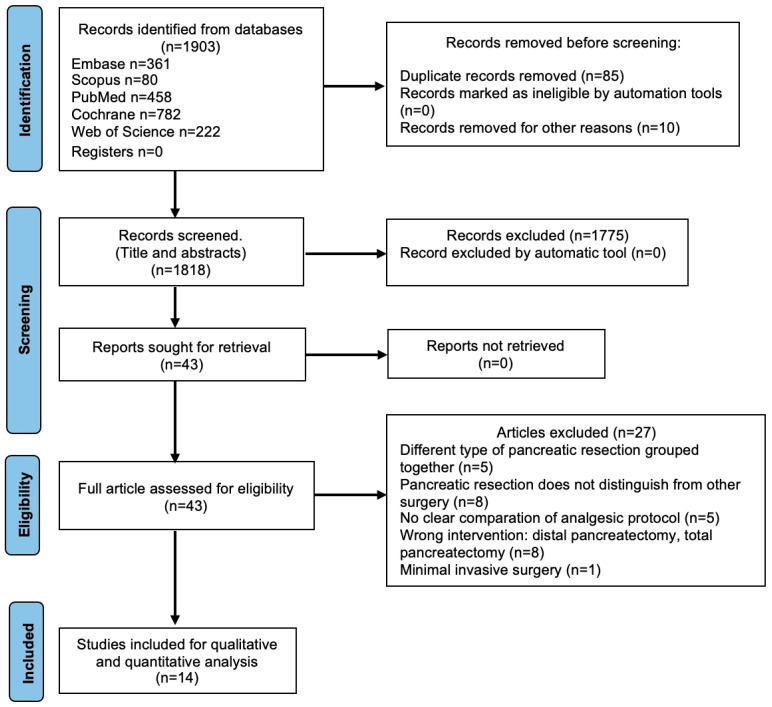
PRISMA flow diagram of the screening process.

The literature search identified 1903 studies. After duplicate removal and screening of titles and abstracts, 1775 studies were excluded because of irrelevance for this study. Details of the screening process are illustrated in Figure 1. Forty-three full-text articles were assessed for eligibility; of these, five RCTs [23,27,35,36,45] and ten retrospective cohort studies were included [10,16,17,18,20,22,24,40,43,44]. The reason for the exclusion of full text is presented in Figure 1.

Finally, 10 studies were included in the meta-analysis [10,16,17,18,22,23,24,27,40,43] and the remaining 5 studies were included in the narrative review [20,35,36,44,45].

### 3.1. Study Characteristics

Overall, 2556 patients were included who underwent pancreatoduodenectomy. Studies were conducted in the USA (*n* = 9), New Zealand (*n* = 1), Netherlands (*n* = 3), Italy (*n* = 1), and other parts of Europe (*n* = 1) and were published between 2008 and 2022. The characteristics of the studies are detailed in Table 1.

Nine articles compared iv opioid (PCA) analgesia to epidural analgesia (EA) [10,17,18,20,22,23,24,27], and two articles compared ITM followed by iv PCA to EA [40,43] and were included in the quantitative analysis. One RCT compared EA and continuous wound infiltration plus PCA morphine (PCA-CWI) [35] and another RCT [36] compared EA and continuous bilateral thoracic paravertebral infusion (CTPVI). Furthermore, one RCT [45] compared sublingual sufentanil and EA/PCA, and one retrospective study [44] compared iv PCA morphine alone and ITM followed by iv PCA (ITM-PCA) or ITM followed by iv PCA plus TAP (ITM-TAP-PCA).

### 3.2. Risk of Bias Assessment

The results of the risk of bias assessment are presented in Table 2 and Table 3. Concerning randomized controlled studies, four had some concerns regarding the overall risk of bias and one study was at high risk of bias. The most important domains affected by bias were the outcome measurement and the selection of the reported result. Within the cohort studies, three studies [16,18,20] were considered to have an overall high risk of bias and seven studies [10,17,22,24,40,43,44] had a moderate risk of bias. The most frequent observation was a moderate risk of bias due to confounding, measurement in outcomes, and selection of the reported result.

### 3.3. Primary Clinical Outcome

Pain scores on postoperative days 1 and 2.

Seven studies [16,17,18,20,23,24,27] reported the mean pain scores for PCA versus EA and six [16,17,18,23,24,27] were included in the meta-analysis. Five studies [35,36,43,44,45] reported pain scores but were not included in the meta-analysis.

#### 3.3.1. PCA versus EA

**Figure 2:**
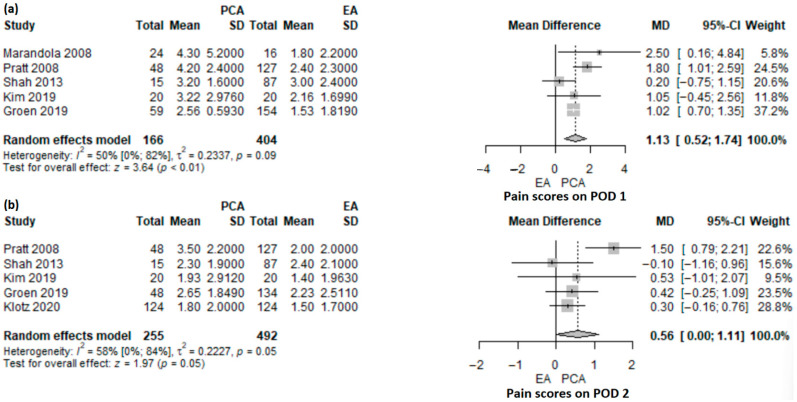
Forest plot of pain scores on POD1 (**a**) and POD2 (**b**) for PCA versus EA following pancreatoduodenectomy. PCA, patient-controlled analgesia; EA, epidural analgesia; SD, standard deviation; MD, mean difference; CI, confidence interval; I^2^, inconsistency index; POD, postoperative day; Pratt et al. [16], Groen et al. [17], Shah et al. [18], Marandola et al. [23], Kim et al. [24], Klotz et al. [27].

Pain score on POD1 was reported in five studies [16,17,18,23,24], including a total of 570 patients (PCA: *n* = 166, EA = 404), of which one study was an RCT [23] and the others were retrospective cohort studies [16,17,18,24]. The mean pain score was significantly higher in the PCA group compared with the EA group (MD 1.13, 95% CI: 0.52–1.74, *p* < 0.001) (Figure 2a). There was a moderate heterogeneity (50%; 95% CI: 0–81.7%), *p* = 0.091) and it was explored with a leave-one-out sensitivity analysis (Supplementary Figure S1). No matter which study was removed, the results were robust and remained statistically significant, pointing in the same direction. Only Marandola et al. [23] had a randomized controlled design, and their results were statistically significant. A sub-analysis of the other studies with a non-randomized design offered an MD of 1.05 (95% CI: 0.42–1.68; *p* = 0.001), which was statistically significant. The randomized study observed higher differences compared with non-randomized designs.

Five studies, one RCT [27] and four retrospective cohort studies [16,17,18,24], included 645 patients (PCA: *n* = 255, EDA: *n* = 492), and reported pain scores on POD2. The mean pain score was higher in the PCA group compared with the EA group, but not statistically significant (MD 0.56; 95% CI: 0–1.11; *p* = 0.05) (Figure 2b). The heterogeneity of studies was statistically significant (58%; 95% CI: 0–84.4%; *p* = 0.05). In the leave-one-out sensitivity analysis (Supplementary Figure S2), the results were not statistically significant anymore, except for the case when the study of Shah et al. [18] was omitted.

One retrospective cohort study [20] reported the median pain score in 42 patients who underwent pancreatoduodenectomy (PCA: 24, EA: 18). They observed no differences in pain score on POD1 (1.8 versus 1.2; *p* = 0.30), but a higher pain score in the PCA group versus the EA group, on POD2 (2.3 versus 1.3; *p* = 0.03).

#### 3.3.2. Regional Techniques versus EA

One RCT [35] compared continuous wound infiltration (CWI) with local anesthetic added to PCA morphine to EA in 36 patients who underwent pancreatoduodenectomy (CWI-PCA: 18, EA:18). The pain scores were lower and similar in both groups on POD1 (1.75 ± 1.26 versus 1.2 ± 0.45; *p* = 0.08) and POD2 (0.75 ± 1.5 versus 1.2 ± 1.1; *p* = 0.3). In an RCT of 48 patients [36], comparing bilateral paravertebral continuous infusion and epidural infusion, there was no significant difference in the maximum pain score expressed as the median and range on both POD1 (7 (3–10) versus 6 (4–10), *p* = 0.67) and POD2 (5 (0–10) versus 5 (2–8), *p* = 0.92).

#### 3.3.3. Sublingual Sufentanyl (SST) versus EA

An RCT [45] of 21 patients undergoing open pancreatoduodenectomy compared sufentanil sublingual (STT:10) and EA (as PCEA:10) and showed similar mean pain scores on postoperative day 1 to 3 (MD, −0.23; 95% CI: −1.22–0.75).

#### 3.3.4. Intrathecal Morphine versus EA

**Figure 3:**
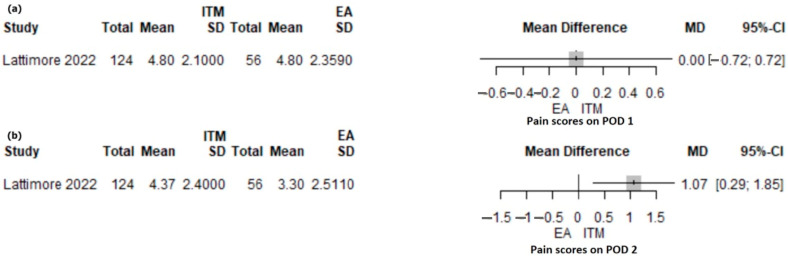
Forest plot for pain scores on POD1 (**a**) and POD2 (**b**) with ITM versus EA following PD. ITM, intrathecal morphine; EA, epidural analgesia; MD, mean difference; SD, standard deviation; CI, confidence interval; I2, inconsistency index; POD, postoperative day; Lattimore et al. [43].

A retrospective study of 180 patients [43] compared intrathecal morphine (ITM: 124) and thoracic epidural analgesia (EA: 56). The mean pain score on POD1 was similar for the ITM and EA groups (MD 0.0; 0.95% CI: −0.72–0.72, *p* = 1) (Figure 3a). On POD2, the mean pain score was significantly higher in the ITM group compared with the EA group (MD 1.07; 95% CI: 0.29–1.85; *p* = 0.007) (Figure 3b). ITM patients received PCA morphine in 40.7% of patients, intravenous lidocaine in 73.4% of patients, and abdominal wall block in 23.4% of patients.

#### 3.3.5. PCA versus ITM Followed iv PCA (ITM-PCA) or versus ITM Followed iv PCA Plus Transversus Abdominis Block (ITM-TAP-PCA)

Burchard et al. [44] compared pain scores in 233 patients following pancreaticoduodenectomy who received PCA alone (PCA:85) or ITM followed by iv PCA (ITM-PCA:155) or ITM followed by iv PCA plus TAP (ITM-TAP-PCA: 33). The average pain score on POD 0 to 3 expressed as median (IQR) was similar between groups as follows: PCA 2.8 (2.0–4.5), ITM-PCA 2.6 (1.6–4.1), and ITM-TAP-PCA 2.3 (1.3–3.4).

### 3.4. Secondary Clinical Outcomes

#### 3.4.1. Duration of Hospital Stay

Length of stay (LOS) was reported in 1918 patients included in seven studies. Five studies [10,16,17,24,27] comparing PCA to EA (1701 patients) and two studies [40,43] comparing ITM to EA (217 patients) were included in meta-analysis.

**Figure 4:**
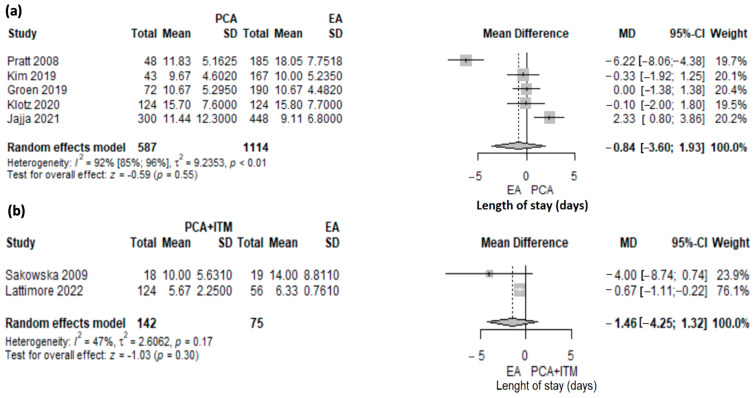
Forest plot for length of stay (LOS) with PCA versus EA (**a**) and PCA + ITM versus EA (**b**) following PD. PCA, patient-controlled analgesia; EA, epidural analgesia; ITM, intrathecal morphine, MD, mean difference; SD, standard deviation; CI, confidence interval; I2, inconsistency index; Jajja et al. [10], Pratt et al. [16], Groen et al. [17], Kim et al. [24], Klotz et al. [27], Sakowska et al. [40], Lattimore et al. [43].

The length of stay was shorter in PCA compared with EA but not statistically significant (MD 0.84; 95% CI: −1.93 to 3.6, *p* = 0.55) (Figure 4a). A significant heterogeneity was found (I2 92.2%, 95% CI: 84.7–96%, *p* < 0.001). The heterogeneity was explored with a leave-one-out sensitivity analysis (Supplementary Figure S3). The study of Pratt [16] was an outlier. The data in this study were presented as the median and range, with the maximum value being very high. Based on these values, we computed the mean and standard deviation, and the values were influenced by this. When removing the study of Pratt [16] from the analysis, the length of stay was longer in the PCA group compared with the EA group (MD 0.50; 95% CI: −75–1.74, I2 = 60%.) Any other removal during the sensitivity analyses did not reduce heterogeneity substantially.

There was no significant difference between ITM and EA (MD 1.46, 95% CI: −1.32–4.25, *p* = 0.3) (Figure 4b). A moderate heterogeneity was found, albeit not statistically significant (I2 = 47%, *p* = 0.17).

Another retrospective study [44] of 233 patients, comparing PCA alone (85 patients) to ITM followed by iv PCA (ITM-PCA) (115 patients) or ITM followed by iv PCA plus TAP block (ITM-TAP-PCA) (33 patients), reported a significant reduction in the median (IQR) hospital length of stay for ITM-PCA or ITM-TAP-PCA versus PCA alone (7 days, IQR: 6–11 versus 9 days, IQR: 7–14) (*p* < 0.0001). There was no significant difference regarding LOS between EA and bilateral thoracic paravertebral continuous infusion [36].

#### 3.4.2. Specific Complications: Postoperative Pancreatic Fistula, Bile Leakage, Delayed Gastric Emptying, Ileus, and Gastrointestinal Bleeding

**Figure 5:**
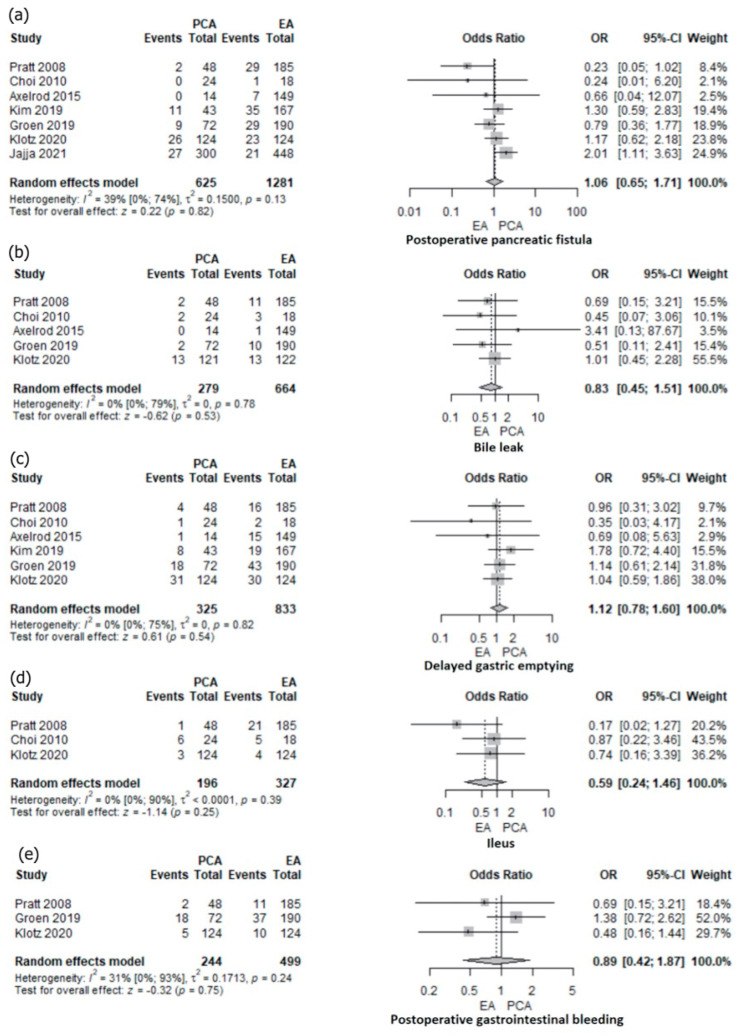
Forest plot for specific postoperative complications: pancreatic fistula (**a**), bile leak (**b**), delayed gastric emptying (**c**), ileus (**d**), and gastrointestinal bleeding (**e**) with PCA versus EA following PD; Jajja et al. [10], Pratt et al. [16], Groen et al. [17], Choi et al. [20], Axelrod et al. [22], Kim et al. [24], Klotz et al. [27].

The analysis of postoperative complications such as pancreatic fistula was reported in six retrospective studies [10,16,17,20,22,24] and one RCT [27]. No significant difference was observed between PCA and EA (OR 1.06; 95% CI: 0.65–1.71; *p* = 0.82) (Figure 5a). The heterogeneity was low and not statistically significant (I2 = 39%, *p* = 0.13).

The bile leakage was analyzed in one RCT [27] and four non-randomized studies [16,17,20,22], and no significant difference was identified between PCA and EA (OR 0.83; 95% CI: 0.45–1.51; *p* = 0.53; I2 0%) (Figure 5b). Delayed gastric emptying (DGE) was reported in six studies, one RCT [27] and five cohort studies [16,17,20,22,24], and no differences were reported between PCA and EA (OR 1.12; 95% CI: 0.78–1.6; *p* = 0.54, I2 0%) (Figure 5c). There was no difference in postoperative ileus (OR 0.59; 95% CI: 0.24–1.46; *p* = 0.25; I2 0%) (Figure 5d) and gastrointestinal bleeding (OR 0.89; 95% CI: 0.42–1.87; *p* = 0.75) (Figure 5e) between PCA and EA. Low heterogeneity (I2 30.8%, *p* = 0.23) was found in the gastrointestinal bleeding analysis. One RCT [27] and two non-randomized studies were included in the ileus [16,20] and gastrointestinal bleeding [16,17] analysis. Subgroup analysis of randomized and non-randomized studies was not possible.

### 3.5. Risk of Bias across Studies

The Egger test, for all analyses, had *p*-values above the threshold of significance.

## 4. Discussions

The present systematic review and meta-analysis of analgesic management in patients undergoing pancreatoduodenectomy demonstrated that intravenous opioid analgesia (PCA) provided higher pain scores on postoperative day 1 compared with epidural analgesia. However, when continuous wound infiltration was added to opioid systemic analgesia (PCA morphine) and compared to epidural analgesia, no significant differences in pain scores were demonstrated. Furthermore, there were no significant differences in pain scores when comparing intrathecal morphine followed by iv PCA to EA for the first postoperative day. Continuous bilateral paravertebral infusion (CTPVI) provided similar pain relief as epidural analgesia on POD1 and POD2. The surgical complications and length of hospital stay were not significantly different according to the type of analgesia. LOS was reduced when comparing ITM followed by iv opioid PCA to PCA opioid alone.

In major abdominal surgery, and more relevant in upper gastrointestinal and hepato-pancreato-biliary surgery, where procedures can be disabling and most patients undergo oncologic resection, poorly controlled postoperative pain is associated with increased morbidity, delayed functional recovery, prolonged hospital stays, and reduced quality of life [3,4,5].

Thoracic epidural analgesia was considered for decades as a gold standard for pain relief following open major abdominal surgery, providing a reduction in rest pain, but the evidence regarding the impact on dynamic pain was not conclusive [12,25,28]. A meta-analysis of RCTs [28] comparing epidural analgesia to other alternative analgesic techniques (mainly IV opioids) in major abdominal surgery showed that epidural analgesia might be associated with superior rest pain control in the first 24–48 postoperative hours. On the contrary, a recent Cochrane review [12] reported, in abdominal surgery, a modest reduction in rest pain in the first 24 h after surgery with the use of EA versus opioid PCA. However, in terms of movement, epidural analgesia provides a greater reduction in pain scores compared with systemic opioids.

In our meta-analysis following pancreatoduodenectomy, when comparing intravenous opioid analgesia (PCA) to epidural analgesia (EA), we looked at the effectiveness and safety of analgesia for the first and second days after surgery. Accordingly, higher pain scores were reported in PCA on both days, but the clinical relevance of the superiority of EA analgesia was reduced. The largest mean differences in pain scores (MD 1.13, 95% CI: 0.52–1.74, *p <* 0.001) between PCA and EA were observed in POD1 and can be considered to have some clinical relevance. On POD2, the mean differences in pain scores between PCA and EA were lower (MD 0.56, 95% CI: 0–1.11, *p* = 0.05) and can be considered of limited clinical relevance.

Marandola et al. [23], in an RCT including 40 patients who underwent pancreatoduodenectomy, demonstrated better pain relief on the first postoperative day in epidural analgesia compared with intravenous opioid analgesia. Klots et al. [27], in a multicenter RCT including 248 patients following pancreatoduodenectomy, reported low pain scores on the second postoperative day in both groups PCA versus EA and, in consequence, the effectiveness of both procedures.

Our results are similar to those presented by Groen et al. [56] in their meta-analysis of epidural analgesia in patients following pancreatoduodenectomy. Patients receiving EA had slightly lower pain scores (MD −0.50; 95% CI: −0.8 to −0.21; *p* < 0.001) on days 0–3 compared with intravenous opioid analgesia. A significant difference in pain score was reported on POD1 (MD −1.08; 95% CI: −1.66 to −0.55; *p* < 0.001). In addition, another meta-analysis by Akter et al. [57] regarding analgesic modalities in pancreatic resection showed that EA compared with PCA in pancreatoduodenectomy provides a similar level of postoperative pain relief on POD2 (MD −0.29; 95% CI: −0.83 to −0.25; *p* = 0.04).

The general benefits of epidural infusion in pancreatic surgery, regarding better postoperative pain scores than intravenous analgesia, are observed in the first two postoperative days in most of the studies [1,9,10,15,20,23,24,27]; however, they are diminished further on.

Despite improved analgesia, the clinical benefits of epidural analgesia in terms of reduced postoperative morbidity (pulmonary, cardiovascular, thromboembolic, infectious, and surgical complications) and, in consequence, the length of hospital stay (LOS) showed conflicting evidence in different studies [1,11,12,13,15,21,24,28,58]. Patients on EA require increased perioperative fluid administration and vasopressors, which could influence the outcome.

In our study, we did not find any association between PCA or EA and gastrointestinal complications after pancreatoduodenectomy (pancreatic fistula, bile leakage, delayed gastric emptying, ileus, and gastrointestinal bleeding). Similar results regarding pancreatic fistula, ileus, bile leak, and delayed gastric emptying were reported by other meta-analyses [56,57] in patients receiving PCA or EA following pancreatoduodenectomy.

Regarding LOS, our analysis did not find a significant difference between PCA and EA (MD 0.84, 95% CI: −1.93–3.6, *p* = 0.55), but LOS was slighter longer in PCA when the study of Pratt et al. [16] was omitted from the analysis. The present evidence regarding the impact of pain management on the length of stay in pancreaticoduodenectomy is derived predominantly from several retrospective studies [10,16,17,24] and only one RCT [27] with significant heterogeneity between studies (I2 92.2%, *p <* 0.001). The RCT of Klots et al. [27] showed a similar duration of hospital stay (15.7 ± 7.6 days) in PCA and EA groups, knowing that there were no differences in gastrointestinal or other complications (infectious, pulmonary, cardiovascular, and renal) between the groups. On the contrary, a retrospective study by Jajja et al. [10] reported a significant difference in LOS in PCA compared with EA (11.44 ± 12.3 versus 9.11 ± 6.8 days, *p* = 0.003) in 748 patients that underwent pancreatoduodenectomy. On a multivariate logistic regression analysis of the predictors for LOS, the authors [10] reported the use of PCA as being a significant predictor of LOS alongside ileus and delayed gastrointestinal emptying (*p* < 0.001). Our results were comparable to those reported by Akter et al. [57] in their meta-analysis when comparing PCA to EA in a subgroup of patients following pancreatoduodenectomy—no significant differences in LOS between PCA and EA (MD 0.09; 95% CI: −0.25 to 0.42; *p* = 0.38; I2 0%) were shown. However, a significantly shorter LOS after pancreatoduodenectomy, in EA compared with PCA (MD −2.69; 95% CI: −2.76 to −2.62; *p* < 0.001; I2 99%), was reported by Groen et al. [56] in their meta-analysis, based on only two retrospective studies with a very high heterogeneity between studies.

Very attractive analgesic opportunities in open hepato-biliary-pancreatic surgery are provided by transabdominal wall catheters (TAWCs) with continuous infusion of local anesthetic as a part of a multimodal analgesic regimen. TAWCs, compared with epidural analgesia, provide a similar level of pain relief but with fewer complications [31,32,34,37]. Continuous bilateral thoracic paravertebral infusion provided similar pain relief to epidural analgesia in major abdominal surgery, but with less side effects [59,60], and appeared to be a promising analgesic technique in terms of efficacy and safety [33].

In the present review, one RCT [35] compared EA to CWI plus PCA, and one RCT [36] compared EA to continuous bilateral thoracic paravertebral infusion following pancreatoduodenectomy. Thus, Mungroop et al. [35] compared EA to continuous wound infusion (CWI) plus PCA morphine (CWI-PCA) in a subgroup of 36 patients undergoing pancreatoduodenectomy. The authors reported a non-inferiority of CWI versus EA regarding pain scores, patient satisfaction, and complications. CWI-PCA was associated with a lower mean cumulative vasopressor and opioid consumption. Hutchins et al. [36] compared EA to continuous bilateral thoracic paravertebral infusion (CTPVI) in patients undergoing pancreatoduodenectomy, and they found a similar pain score on POD1 and POD2.

A non-inferiority of continuous wound infusion plus opioid PCA versus epidural analgesia in terms of pain relief, length of hospital stays, and opioid consumption was reported by Spoto et al. [61] in an RCT that included 80 patients who underwent pancreatic resection (pancreatectomy over 70%). However, further randomized large trials are needed for a definitive conclusion regarding the role of TAWCs and paravertebral block in pancreatoduodenectomy as a new strategy for effective pain relief in the perioperative period with reduced opioid consumption and improved functional recovery. The use of wound catheters as an alternative to epidural analgesia is recommended by the ERAS guidelines of perioperative care for pancreatoduodenectomy [6].

Regarding the efficacy and safety of ITM compared with EA in open hepato-pancreato-biliary surgery, a recent review [41] showed that the pain scores were lower or similar to EA in the first 24 h post-surgery and the length of hospital stay was reduced. In our review, one retrospective study [43] compared the analgesic efficacy of ITM as a part of multimodal pain management to EA in pancreatoduodenectomy and reported no difference in mean pain score on POD1, but significantly higher pain scores were reported on POD2 in the ITM group. However, no significant difference in postoperative pain score (over five days), opioid use, and length of hospital stay was reported between ITM and EA. When ITM followed by iv opioid PCA was compared to PCA alone after pancreatoduodenectomy, one study [44] reported a shorter length of hospital stays in the ITM group.

Sublingual sufentanil (SST) showed promising results as an analgesic option following pancreatoduodenectomy when compared with iv opioid PCA or patient-controlled epidural analgesia [45]. In this first randomized study [45] on a subgroup of 21 patients who underwent pancreatoduodenectomy, similar pain scores were reported on postoperative days 1 to 3 between SST and PCA or PCEA.

There are some limitations of the present review and meta-analysis. Most of the included studies were small, retrospective, non-randomized, single-center trials; although we included five RCTs in the narrative review, only two could be included in our meta-analysis. Regarding the risk of bias, the randomized controlled trials had some concerns of bias, and one trial had a high risk of bias. The observational studies included in our meta-analysis had a moderate risk of bias, except three studies that had a serious risk of bias. The postoperative pain scores and the specific gastrointestinal complications were not assessed in all studies; thereby, a meta-analysis was possible for opioid PCA versus epidural analgesia. The pain scores with movement were not reported. In our study, for several outcomes, we found the between-study heterogeneity to range from moderate to high, being statistically significant in the pain scores on the POD2 comparison and the length of stay comparison. We explored this heterogeneity with posthoc leave-one-out sensitivity analyses and, in the case of pain on POD1, the results were robust. Because of the limited data available regarding the opioid-sparing strategy analgesia (EA versus PCA plus continuous wound infiltration with local anesthetic) and the intrathecal opioid analgesia in pancreatoduodenectomy, the meta-analysis of primary or secondary outcomes was not possible and was limited to a narrative review.

For clinicians, the present review and meta-analysis summarized the available evidence regarding pain management following pancreatoduodenectomy. Most of the evidence is centered around the efficacy and safety of EA, PCA, and other opioid-sparing strategies such as continuous wound infusion or continuous bilateral continuous paravertebral. In addition, ITM, as a part of multimodal analgesia, has emerged as a promising alternative to epidural infusion in pancreatic surgery, offering similar analgesia with an enhanced safety profile.

More large randomized controlled trials that pay close attention to limit their biases are needed to evaluate the real advantages and disadvantages of these analgesic techniques in pancreatoduodenectomy, not only on postoperative pain, but on postoperative outcomes in the settings of ERAS pathways.

## 5. Conclusions

EA provided lower pain scores compared with PCA on the first postoperative day after pancreatoduodenectomy; the length of stay and procedure-related complications were similar between EA and PCA. Continuous wound infusion added to opioid PCA, and continuous bilateral thoracic paravertebral infusion provided similar pain relief compared with EA in the first two postoperative days after pancreatoduodenectomy.

## Figures and Tables

**Table 1:** Study characteristics.

	Publication Year	Country	Recruitment Dates	Centers	Type of Pancreatic Resection	Analgesic Modalities Compared (*n*)	
RCTs							
Klotz et al. [27]	2020	Europe	2015–2017	9	PD	PCA (124)	EA (124)
Mungroop et al. [35]	2016	NL	2015	2	PD	CWI-PCA (18)	EA (18)
Hutchins et al. [36]	2018	USA	2012–2015	1	PD	TPVB (25)	EA (23)
Marandola et al. [23]	2008	Italy	2002–2007	1	PD	PCA (24)	EA (16)
Groen et al. [45]	2022	NL	2018–2021	1	PD	SST (10)	EA-PCEA (10) /PCA (1)
Cohort studies							
Burchartd et al. [44]	2022	USA	2014–2020	1	PD	PCA (85)	PCA/ITM (115) PCA/ITM/TAP (33)
Lattimore et al. [43]	2022	USA	2015–2020	1	PD	ITM-PCA (124)	EA (58)
Jajja et al. [10]	2021	USA	2010–2017	1	PD	PCA (300)	EA (448)
Kim et al. ** [24]	2019	USA	2013–2016	1	PD	PCA (43)	EA (167)
Groen et al. * [17]	2019	NL	2013–2017	1	PD	PCA (59)	EA (154)
Axelrot et al. [22]	2015	USA	2007–2011	1	PD	PCA (14)	EA (149)
Shah et al. [18]	2013	USA	2007–2011	1	PD	PCA (15)	EA (87)
Choi et al. [20]	2010	USA	2004–2007	1	PD	PCA (24)	EA (18)
Sakowska et al. [40]	2009	NZ	2005–2008	1	PD	PCA-ITM (18)	EA (19)
Pratt et al. [16]	2008	USA	2001–2007	1	PD	PCA (48)	EA (185)

RCT, randomized controlled trial; NL, Netherlands; NZ, New Zealand; PD, pancreatoduodenectomy; PCA, patient-controlled analgesia; EA, epidural analgesia; PCEA, patient controlled epidural analgesia; ITM, intrathecal morphine; CWI, continuous wound infusion; TAP, transverse abdominis plane block; TPBV, thoracic paravertebral block; SST, sublingual sufentanil. * Groen et al. [17]—74.7% pancreatoduodenectomy. ** Kim et al. [24]—sub analysis for pain score comparison.

**Table 2:** Risk of bias for RCTs according to the Cochrane Collaboration tool 2.0.

	Randomization Process	Deviations from the Intended Interventions	Missing Outcome Data	Measurement of the Outcome	Selection of the Reported Result	Overall Risk of Bias
Klotz et al. [27] 2020	Low risk	Low risk	Low risk	Some concern	Low risk	Some concern
Mungroop et al. [35] 2016	Low risk	Low risk	Low risk	Some concern	Low risk	Some concern
Marandola et al. [23] 2008	High risk	High risk	Low risk	Some concern	Some concern	High risk
Groen et al. [45] 2022	Low risk	Some concern	Low risk	Some concern	Low risk	Some concern
Hutchins et al. [36] 2018	Low risk	Low risk	Low risk	Some concern	Some concern	Some concern

**Table 3:** Risk of bias for cohort studies according to the Robins-I tool.

	Bias Due to Confounding	Bias in Selection of Participants	Bias in Classification of Interventions	Bias Due to Deviations from Intended Interventions	Bias Due to Missing Data	Bias in Measurement of Outcomes	Bias in Selection of the Reported Result	Overall Bias
Burchartd et al. [44] 2022	moderate	low	low	low	low	moderate	moderate	moderate
Lattimore et al. [43] 2022	moderate	low	low	low	low	moderate	moderate	moderate
Jajja et al. [10] 2021	moderate	low	low	low	low	moderate	moderate	moderate
Kim et al. [24] 2019	moderate	low	low	low	low	moderate	moderate	moderate
Groen et al. [17] 2019	moderate	low	low	low	low	moderate	moderate	moderate
Axelrot et al. [22] 2015	moderate	low	low	low	low	moderate	moderate	moderate
Shah et al. [18] 2013	moderate	low	low	low	low	serious	moderate	serious
Choi et al. [20] 2010	serious	low	low	low	low	serious	moderate	serious
Sakowska et al. [40] 2009	moderate	low	low	low	low	low	moderate	moderate
Pratt et al. [16] 2008	moderate	low	low	moderate	low	serious	moderate	serious

## Data Availability

Not applicable.

## Supplementary Materials

The following supporting information can be downloaded at https://www.mdpi.com/article/10.3390/jcm12144682/s1. Supplementary Materials S1: Appendix S1—Search literature strategy; Supplementary Materials S2: Figure S1—Leave-one-out sensitivity analysis plot for pain on POD1, Figure S2—Leave-one-out sensitivity analysis plot for pain on POD2, Figure S3—Leave-one-out sensitivity analysis plot for length of stay.

## References

[1] Aloia T.A., Kim B.J., Segraves-Chun Y.S., Cata J.P., Truty M.J., Shi Q., Holmes A., Soliz J.M., Popat K.U., Rahlfs T.F. (2017). A Randomized Controlled Trial of Postoperative Thoracic Epidural Analgesia Versus Intravenous Patient-controlled Analgesia After Major Hepatopancreatobiliary Surgery. Ann. Surg..

[2] Newhook T.E., Dewhurst W.L., Vreeland T.J., Wang X., Soliz J., Speer B.B., Hancher-Hodges S., Feng C., Bruno M.L., Kim M.P. (2019). Inpatient Opioid Use After Pancreatectomy: Opportunities for Reducing Initial Opioid Exposure in Cancer Surgery Patients. Ann. Surg. Oncol..

[3] Kehlet H., Holte K. (2001). Effect of postoperative analgesia on surgical outcome. Br. J. Anaesth..

[4] Rawal N. (2016). Current issues in postoperative pain management. Eur. J. Anaesthesiol..

[5] Pirie K., Traer E., Finniss D., Myles P.S., Riedel B. (2022). Current approaches to acute postoperative pain management after major abdominal surgery: A narrative review and future directions. Br. J. Anaesth..

[6] Melloul E., Lassen K., Roulin D., Grass F., Perinel J., Adham M., Wellge E.B., Kunzler F., Besselink M.G., Asbun H. (2020). Guidelines for perioperative care for pancreatoduodenectomy: Enhanced Recovery After Surgery (ERAS) Recommendations 2019. World J. Surg..

[7] Rigg J.R., Jamrozik K., Myles P.S., Silbert B.S., Peyton P.J., Parsons R.W., Collins K.S., MASTER Anaethesia Trial Study Group (2002). Epidural anaesthesia and analgesia and outcome of major surgery: A randomised trial. Lancet.

[8] Fotiadis R.J., Badvie S., Weston M.D., Allen-Mersh T.G. (2004). Epidural analgesia in gastrointestinal surgery. Br. J. Surg..

[9] Simpson R.E., Fennerty M.L., Colgate C.L., Kilbane E.M., Ceppa E.P., House M.G., Zyromski N.J., Nakeeb A., Schmidt C.M. (2019). Post-Pancreaticoduodenectomy Outcomes and Epidural Analgesia: A 5-year Single-Institution Experience. J. Am. Coll. Surg..

[10] Jajja M.R., Williams H., Mahmooth Z., Nadeem S.O., Hashmi S.S., Sarmiento J.M. (2022). Narcotic sparing postoperative analgesic strategies after pancreatoduodenectomy: Analysis of practice patterns for 1004 patients. HPB.

[11] Shi W.Z., Miao Y.L., Yakoob M.Y., Cao J.B., Zhang H., Jiang Y.G., Xu L.H., Mi W.D. (2014). Recovery of gastrointestinal function with thoracic epidural vs. Systemic analgesia following gastrointestinal surgery. Acta Anaesthesiol. Scand..

[12] Salicath J.H., Yeoh E.C., Bennett M.H. (2018). Epidural analgesia versus patient-controlled intravenous analgesia for pain following intra-abdominal surgery in adults. Cochrane Database Syst. Rev..

[13] Guay J., Nishimori M., Kopp S. (2016). Epidural local anaesthetics versus opioid-based analgesic regimens for postoperative gastrointestinal paralysis, vomiting and pain after abdominal surgery. Cochrane Database Syst. Rev..

[14] Volk T., Wolf A., Van Aken H., Burkle H., Wiebalck A., Steinfeldt T. (2012). Incidence of spinal haematoma after epidural puncture: Analysis from German network for safety in regional anaesthesia. Eur. J. Anaesthesiol..

[15] Hermanides J., Hollmann M.W., Stevens M.F., Lirk P. (2012). Failed epidural: Causes and management. Br. J. Anaesth..

[16] Pratt W.B., Steinbrook R.A., Maithel S.K., Vanounou T., Callery M.P., Vollmer C.M. (2008). Epidural analgesia for pancreatoduodenectomy: A critical appraisal. J. Gastrointest. Surg..

[17] Groen J.V., Slotboom D.E.F., Vuyk J., Martini C.H., Dahan A., Vahrmeijer A.L., Bonsing B.A., Mieog J.S.D. (2019). Epidural and non-epidural analgesia in patients undergoing open pancreatectomy: A retrospective cohort study. J. Gastrointest. Surg..

[18] Shah D.R., Brown E., Russo J.E., Li C.S., Martinez S.R., Coates J.M., Bold R.J., Canter R.J. (2013). Negligible effect of perioperative epidural analgesia among patients undergoing elective gastric and pancreatic resections. J. Gastrointest. Surg..

[19] Patel A., Stasiowska M., Waheed U., Brett S.J., Patel P.B. (2014). Poor analgesic efficacy of epidural analgesia in critical care patients after pancreaticoduodenectomy. Pancreas.

[20] Choi D.X., Schoeniger L.O. (2010). For patients undergoing pancreatoduodenectomy, epidural anesthesia and analgesia improves pain but increases rates of intensive care unit admissions and alterations in analgesics. Pancreas.

[21] Sugimoto M., Nesbit L., Barton J.G., Traverso W.L. (2016). Epidural anesthesia dysfunction is associated with postoperative complications after pancreatectomy. J. Hepatobiliary Pancreat. Sci..

[22] Axelrod T.M., Mendez B.M., Abood G.J., Sinacore J.M., Aranha G.V., Shoup M. (2015). Perioperative epidural may not be the preferred form of analgesia in select patients undergoing pancreaticoduodenectomy. J. Surg. Oncol..

[23] Marandola M., Cilli T., Alessandri F., Tellan G., Caronna R., Chirletti P., Delogu G. (2008). Perioperative management in patients undergoing pancreatic surgery: The anesthesiologist’s point of view. Transpl. Proc..

[24] Kim S.S., Niu X., Elliott I.A., Jiang J.P., Dann A.M., Damato L.M., Chung H., Girgis M.D., King J.C., Hines O.J. (2019). Epidural analgesia improves postoperative pain control but impedes early discharge in patients undergoing pancreatic surgery. Pancreas.

[25] Pöpping D.M., Elia N., Van Aken H.K., Marret E., Schug S.A., Kranke P., Wenk M., Tramèr M.R. (2014). Impact of epidural analgesia on mortality and morbidity after surgery: Systematic review and meta-analysis of randomize controlled trials. Ann. Surg..

[26] Negrini D., Ihsan M., Freitas K., Pollazzon C., Graaf J., Andre J., Linhares T., Brandao V., Silva G., Fiorelli R. (2022). The clinical impact of the perioperative epidural anesthesia on surgical outcomes after pancreaticoduodenectomy: A retrospective cohort study. Surg. Open. Sci..

[27] Klotz R., Larmann J., Klose C., Bruckner T., Benner L., Doerr-Harim C., Tenckhoff S., Lock J.F., Brede E.M., Salvia R. (2020). Gastrointestinal Complications After Pancreatoduodenectomy with Epidural vs. Patient-Controlled Intravenous Analgesia: A Randomized Clinical Trial. JAMA Surg..

[28] Hughes M.J., Ventham N.T., McNally S., Harrison E., Wigmore S. (2014). Analgesia after open abdominal surgery in the setting of enhanced recovery surgery: A systematic review and meta-analysis. JAMA Surg..

[29] Roughead E.E., Lim R., Ramsay E., Moffat A.K., Pratt N.L. (2019). Persistence with opioids post discharge from hospitalisation for surgery in Australian adults: A retrospective cohort study. BMJ Open.

[30] Grandhi R.K., Lee S., Abd-Elsayed A. (2017). Does opioid use cause angiogenesis and metastasis?. Pain Med..

[31] Ventham N.T., Hughes M., O’Neill S., Johns N., Brady R.R., Wigmore S.J. (2013). Systematic review and meta-analysis of continuous local anaesthetic wound infiltration versus epidural analgesia for postoperative pain following abdominal surgery. Br. J. Surg..

[32] Mungroop T.H., Bond M.J., Lirk P., Busch O.R., Hollmann M.W., Veelo D.P., Besselink M.G. (2019). Preperitoneal or subcutaneous wound catheters as alternative for epidural analgesia in abdominal surgery: A systematic review and meta-analysis. Ann. Surg..

[33] El-Boghdadly K., Madjdpour C., Chin K.J. (2016). Thoracic paravertebral blocks in abdominal surgery—A systematic review of randomized controlled trials. Br. J. Anaesth..

[34] Jackson A.C., Memory K., Issa E., Isherwood J., Graff-Baker P., Garcea G. (2022). Retrospective observational study of patient outcomes with local wound infusion vs epidural analgesia after open hepato-pancreato-biliary surgery. BMC Anesthesiol..

[35] Mungroop T.H., Veelo D.P., Busch O.R., van Dieren S., van Gulik T.M., Karsten T.M., de Castro S.M., Godfried M.B., Thiel B., Hollmann M.W. (2016). Continuous wound infiltration versus epidural analgesia after hepato-pancreato-biliary surgery (POP-UP): A randomised controlled, open-label, non-inferiority trial. Lancet Gastroenterol. Hepatol..

[36] Hutchins J.L., Grandelis A.J., Kaizer A.M., Jensen E.H. (2018). Thoracic paravertebral block versus thoracic epidural analgesia for post-operative pain control in open pancreatic surgery: A randomized controlled trial. J. Clin. Anesth..

[37] Perrin J., Ratnayake B., Wells C., Windsor J.A., Loveday B.P.T., MacLennan N., Lindsay H., Pandanaboyana S. (2021). Epidural versus transabdominal wall catheters: A comparative study of outcomes after pancreatic resection. J. Surg. Res..

[38] Koning M.V., Klimek M., Rijs K., Stolker R.J., Heesen M.A. (2020). Intrathecal hydrophilic opioids for abdominal surgery: A meta-analysis, meta-regression, and trial sequential analysis. Br. J. Anaesth..

[39] Meylan N., Elia N., Lysakowski C., Tramer M.R. (2009). Benefit and risk of intrathecal morphine without local anaesthetic in patients undergoing major surgery: Meta-analysis of randomized trials. Br. J. Anaesth..

[40] Sakowska M., Docherty E., Linscott D., Connor S. (2009). A change in practice from epidural to intrathecal morphine analgesia for hepato-pancreato-biliary surgery. World J. Surg..

[41] Tang J.Z.J., Weinberg L. (2019). A literature review of intrathecal morphine analgesia in patients undergoing major open hepato-pancreatic-biliary (HPB) surgery. Anesth. Pain Med..

[42] Dichtwald S., Ben-Haim M., Papismedov L., Hazan S., Cattan A., Matot I. (2017). Intrathecal morphine versus intravenous opioid administration to impact postoperative analgesia in hepato-pancreatic surgery: A randomized controlled trial. J. Anesth..

[43] Lattimore C.M., Kane W.J., Sarosiek B.M., Coleman C.M., Turrentine F.E., Forkin K.T., Bauer T.W., Adams R.B., Zaydfudim V.M. (2022). Efficacy of opioid spinal analgesia for postoperative pain management after pancreatoduodenectomy. HPB.

[44] Burchard P.R., Melucci A.D., Lynch O., Loria A., Dave Y.A., Strawderman M., Schoeniger L.O., Galka E., Moalem J., Linehan D.C. (2022). Intrathecal morphine and effect on opioid consumption and functional recovery after pancreaticoduodenectomy. J. Am. Coll. Surg..

[45] Groen J.V., Boon S.C., Minderhoud M.W., Bonsing B.A., Martini C.H., Putter H., Vahrmeijer A.L., van Velzen M., Vuijk J., Mieog J.S.D. (2022). Sublingual Sufentanil versus standard-of-care (patient-controlled analgesia with epidural Ropivacaine/Sufentanil or intravenous Morphine) for postoperative pain following pancreatoduodenectomy: A Randomized Trial. J. Pain Res..

[46] Ringold F.G., Minkowitz H.S., Gan T.J., Aqua K.A., Chiang Y.K., Evashenk M.A., Palmer P.P. (2015). Sufentanil sublingual tablet system for the management of postoperative pain following open abdominal surgery: A randomized, placebo-controlled study. Reg. Anesth. Pain Med..

[47] Page M.J., McKenzie J.E., Bossuyt P.M., Boutron I., Hoffmann T.C., Mulrow C.D., Shamseer L., Tetzlaff J.M., Akl E.A., Brennan S.E. (2021). The PRISMA 2020 statement: An updated guideline for reporting systematic reviews. BMJ.

[48] Bijur P.E., Latimer C.T., Gallagher E.J. (2003). Validation of a verbally administered numerical rating scale of acute pain for use in the emergency department. Acad. Emerg. Med..

[49] Sterne J.A.C., Savovic J., Page M.J., Elbers R.G., Blencowe N.S., Boutron I., Cates C.J., Cheng H.Y., Corbett M.S., Eldridge S.M. (2019). RoB 2: A revised tool for assessing risk of bias in randomised trials. BMJ.

[50] Sterne J.A., Hernán M.A., Reeves B.C., Savović J., Berkman N.D., Viswanathan M., Henry D., Altman D.G., Ansari M.T., Boutron I. (2016). ROBINS-I: A tool for assessing risk of bias in non-randomised studies of interventions. BMJ.

[51] R Core Team (2021). R: A Language and Environment for Statistical Computing.

[52] Balduzzi S., Rücker G., Schwarzer G. (2019). How to perform a meta-analysis with R: A practical tutorial. Evid. Based Ment. Health.

[53] Web Plot Digitizer. https://automeris.io/WebPlotDigitizer/.

[54] Wan X., Wang W., Liu J., Tong T. (2014). Estimating the sample mean and standard deviation from the sample size, median, range and/or interquartile range. BMC Med. Res. Methodol..

[55] Higgins J.P., Thompson S.G., Deeks J.J., Altman D.G. (2003). Measuring inconsistency in meta-analyses. BMJ.

[56] Groen J.V., Khawar A.A.J., Bauer P.A., Bonsing B.A., Martini C.H., Mungroop T.H., Vahrmeijer A.L., Vuijk J., Dahan A., Mieog J.S.D. (2019). Meta-analysis of epidural analgesia in patients undergoing pancreatoduodenectomy. BJS Open..

[57] Akter N., Ratnayake B., Joh D.B., Chan S.J., Bonner E., Pandanaboyana S. (2021). Postoperative Pain Relief after Pancreatic Resection: Systematic Review and Meta-Analysis of Analgesic Modalities. World J. Surg..

[58] Kone L.B., Maker V.K., Banulescu M., Maker A.V. (2021). Epidural analgesia is associated with prolonged length of stay after open HPB surgery in over 27,000 Patients. J. Gastrointest Surg..

[59] Han Y., Dai Y., Shi Y., Zhang X., Xia B., Ji Q., Yu X., Bian J., Xu T. (2022). Ultrasound-guided paravertebral blockade reduced perioperative opioids requirement in pancreatic resection: A randomized controlled trial. Front Surg..

[60] Schreiber K.L., Chelly J.E., Lang R.S., Abuelkasem E., Geller D.A., Marsh J.W., Tsung A., Sakai T. (2016). Epidural Versus Paravertebral Nerve Block for Postoperative Analgesia in Patients Undergoing Open Liver Resection: A randomized clinical trial. Reg. Anesth. Pain Med..

[61] Spoto M.R., Zito P., Molinari A.F., Capretti G., Gavazzi F., Ridolfi C., Grimaldi S., Pesce S., Allavena P., Zerbi A. (2015). A randomized clinical trial to compare the efficacy of continuous local anesthetic wound infusion with thoracic epidural analgesia in post-operative pain control after pancreatic surgery. Anaesth. Pain. Intensive Care.

