# Supplementary material for: Analgesic Modalities in Patients Undergoing Open Pancreatoduodenectomy—A Systematic Review and Meta-Analysis
Source: J Clin Med. 2023 Jul 14;12(14):4682. doi: 10.3390/jcm12144682 (PMC10380756; doi:10.3390/jcm12144682)
Supplement: Supplementary file 1 [file jcm-12-04682-s001.zip › Supplementary Material S1.pdf]

PUBMED- : 458 references

("ANALGESIA, EPIDURAL"[MESH TERMS] OR "ANALGESIA, PATIENT CONTROLLED"[MESH TERMS] OR "ANESTHESIA AND ANALGESIA"[MESH TERMS] OR "PAIN MEASUREMENT"[MESH TERMS] OR "EPIDURAL ANALGESIA"[TEXT WORD] OR "MULTIMODAL ANALGESIA"[TEXT WORD] OR "OPIOID ANALGESIA"[TEXT WORD] OR "POSTOPERATIVE ANALGESIA"[TEXT WORD] OR "TAP BLOCK*"[TEXT WORD] OR "REGIONAL ANALGESIA"[TEXT WORD] OR "PARAVERTEBRAL BLOCK*"[ALL FIELDS] OR "SPINAL"[TEXT WORD] OR "INTRATHECAL BLOCK*"[TEXT WORD] OR "BLOCK*"[TEXT WORD] OR "NARCOTIC*"[TEXT WORD] OR "CONTINUOUS WOUND INFILTRATION*"[TEXT WORD]) AND ("PANCREATECTOMY"[MESH TERMS] OR "PANCREATICODUODENECTOMY"[MESH TERMS] OR "DUODENOPANCREATECTOM*"[TEXT WORD] OR "PANCREATIC RESECTION*"[TEXT WORD] OR "PANCREATIC SURGER*"[TEXT WORD] OR "WHIPPLE SURGERY"[ALL FIELDS] OR "WHIPPLE RESECTION*"[TEXT WORD]) **FILTERS:** FROM 1995 – 2023

Web of science: - 222 references

Timespan: 1995-01-01 to 2023-10-01 (Publication Date)

"ANALGESIA, EPIDURAL"[MESH] OR "ANALGESIA, PATIENT-CONTROLLED"[MESH] OR "ANESTHESIA AND ANALGESIA"[MESH] OR "PAIN MEASUREMENT"[MESH] OR “EPIDURAL ANALGESIA”[TW] OR “MULTIMODAL ANALGESIA”[TW] OR “OPIOID ANALGESIA”[TW] AND "PANCREATECTOMY"[MESH] OR "PANCREATICODUODENECTOMY"[MESH] OR “DUODENOPANCREATECTOM*”[TW] OR “PANCREATIC RESECTION*”[TW] OR “PANCREATIC SURGER*”[TW] OR "PANCREATECTOMY"[MESH] OR “WHIPPLE SURGERY” OR “WHIPPLE RESECTION*”[TW] MESH: "PANCREATECTOMY"[MESH], "PANCREATICODUODENECTOMY"[MESH]

Cochrane: 782 references

(“ANALGESIA” OR “EPIDURAL” OR “ANALGESIA PACIENT CONTROLLED” OR “ANESTHESIA AND ANALGESIA” OR “PAIN MEASUREMENT” OR “EPIDURAL ANALGESIA “ OR “OPIOID ANALGESIA” OR “MULTIMODAL ANALGESIA” OR “POSTOPERATIVE ANALGESIA” OR “OPIOID “ OR “TAP BLOCK “ OR “REGIONAL ANALGESIA” OR “PARAVERTEBRAL BLOCK” OR “SPINAL” OR “SYSTEMIC OPIOID” OR “NON-OPIOID ANALGESIA” AND “PANCREATECTOMY” OR “PANCREATICODUODENECTOMY” OR “DUODENOPANCREATECTOM” OR “PANCREATIC RESECTION” OR “WHIPPLE SURGERY “OR “WHIPPLE RESECTION” OR “PANCREATIC SURGER”): TI,AB,KW

TI=title word

Ab=abstract word

Kw=key word (Mesh and others)

SCOPUS: 57 references

ALL ("ANALGESIA EPIDURAL" OR "ANALGESIA PATIENT-CONTROLLED" OR "ANESTHESIA AND ANALGESIA" OR "PAIN MEASUREMENT" OR "MULTIMODAL ANALGESIA" OR "OPIOID ANALGESIA" OR "POSTOPERATIVE ANALGESIA" OR "TAP BLOCK " OR "REGIONAL ANALGESIA" OR "PARAVERTEBRAL BLOCK " OR "SPINAL" OR "INTRATHECAL BLOCK " OR "OPIOID" OR "SYSTEMIC OPIOID " OR "NON-OPIOID ANALGESIA" OR "NEURAXIS ANALGESIA" OR "BLOCK " OR "NARCOTIC " OR "CONTINUOUS WOUND INFILTRATION " , "ANALGESIA, PATIENT-CONTROLLED" AND "PANCREATICODUODENECTOMY" OR "DUODENOPANCREATECTOMY " OR "PANCREATIC RESECTION" OR "PANCREATIC SURGERY" OR "WHIPPLE SURGERY" OR "WHIPPLE RESECTION" OR "PANCREATECTOMY")

Embase 361 references

(('ANALGESIA EPIDURAL':TI,AB,KW OR 'ANALGESIA PATIENT-CONTROLLED':TI,AB,KW OR ANESTHESIA:TI,AB,KW) AND ANALGESIA:TI,AB,KW OR 'PAIN MEASUREMENT':TI,AB,KW OR 'MULTIMODAL ANALGESIA':TI,AB,KW OR 'OPIOID ANALGESIA':TI,AB,KW OR 'POSTOPERATIVE ANALGESIA':TI,AB,KW OR 'TAP BLOCK':TI,AB,KW OR 'REGIONAL ANALGESIA':TI,AB,KW OR 'PARAVERTEBRAL BLOCK':TI,AB,KW OR 'SPINAL':TI,AB,KW OR 'INTRATHECAL BLOCK':TI,AB,KW OR 'OPIOID':TI,AB,KW OR 'SYSTEMIC OPIOID':TI,AB,KW OR 'NON-OPIOID ANALGESIA':TI,AB,KW OR 'NEURAXIS ANALGESIA':TI,AB,KW OR 'BLOCK':TI,AB,KW OR 'NARCOTIC':TI,AB,KW OR 'CONTINUOUS WOUND INFILTRATION':TI,AB,KW OR 'ANALGESIA, PATIENT-CONTROLLED':TI,AB,KW) AND ('PANCREATICODUODENECTOMY':TI,AB,KW OR 'DUODENOPANCREATECTOMY':TI,AB,KW OR 'PANCREATIC RESECTION':TI,AB,KW OR 'PANCREATIC SURGERY':TI,AB,KW OR 'WHIPPLE SURGERY':TI,AB,KW OR 'WHIPPLE RESECTION':TI,AB,KW OR 'PANCREATECTOMY':TI,AB,KW) AND [1995-2023]/PY AND ([FEMALE]/LIM OR [MALE]/LIM)

TI=title word

Ab=abstract word

Kw=key word (Mesh and others)

PY= publication year

Lim=limit
