# Supplementary material for: Analgesic Modalities in Patients Undergoing Open Pancreatoduodenectomy—A Systematic Review and Meta-Analysis
Source: J Clin Med. 2023 Jul 14;12(14):4682. doi: 10.3390/jcm12144682 (PMC10380756; doi:10.3390/jcm12144682)
Supplement: Supplementary file 1 [file jcm-12-04682-s001.zip › Supplementary Material S2.pdf]

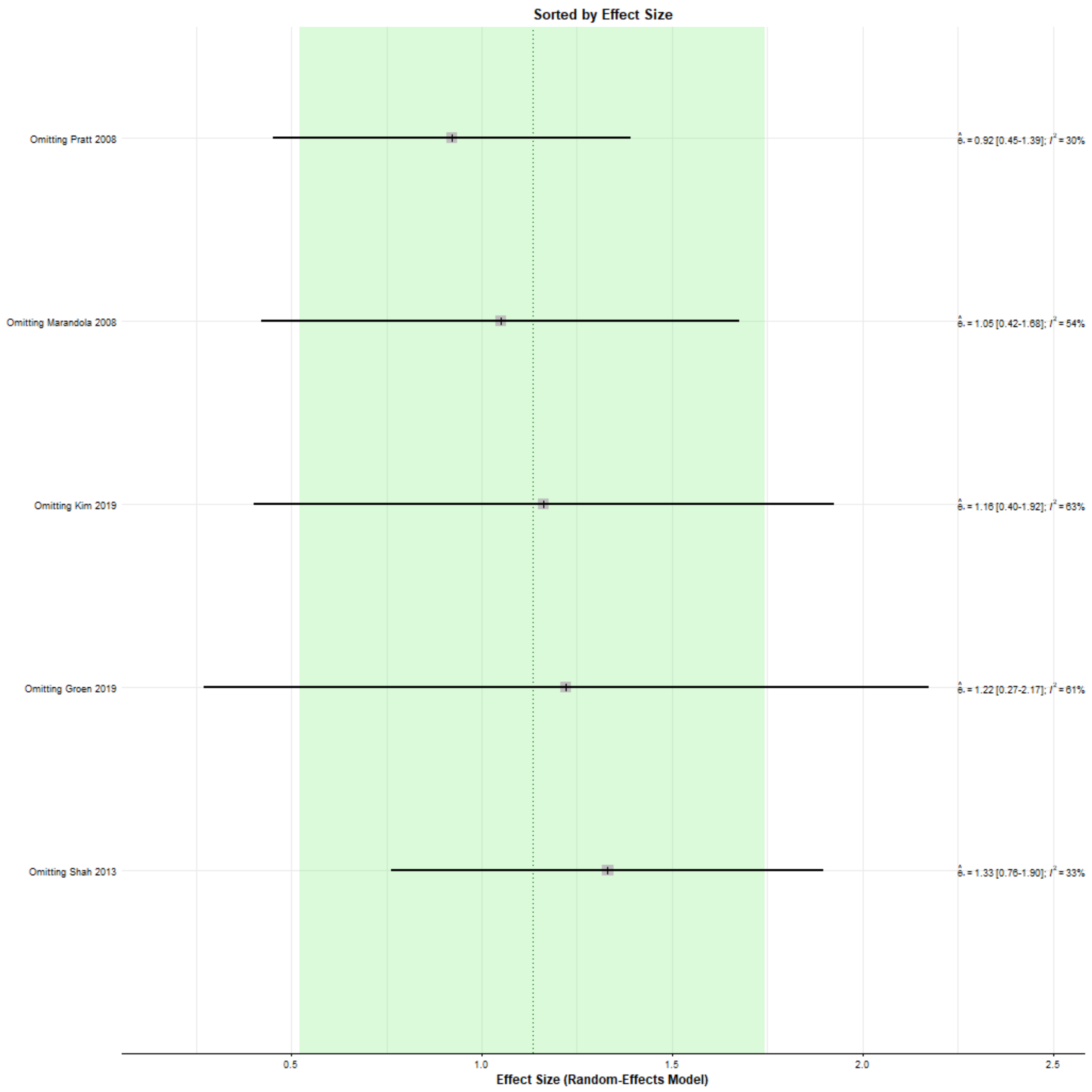

Supplementary Figure S1. Leave-one-out sensitivity analysis plot for selected studies for the pain scores on postoperative day 1 in patient-controlled analgesia versus epidural analgesia.

$\hat{\Theta}$ = mean difference

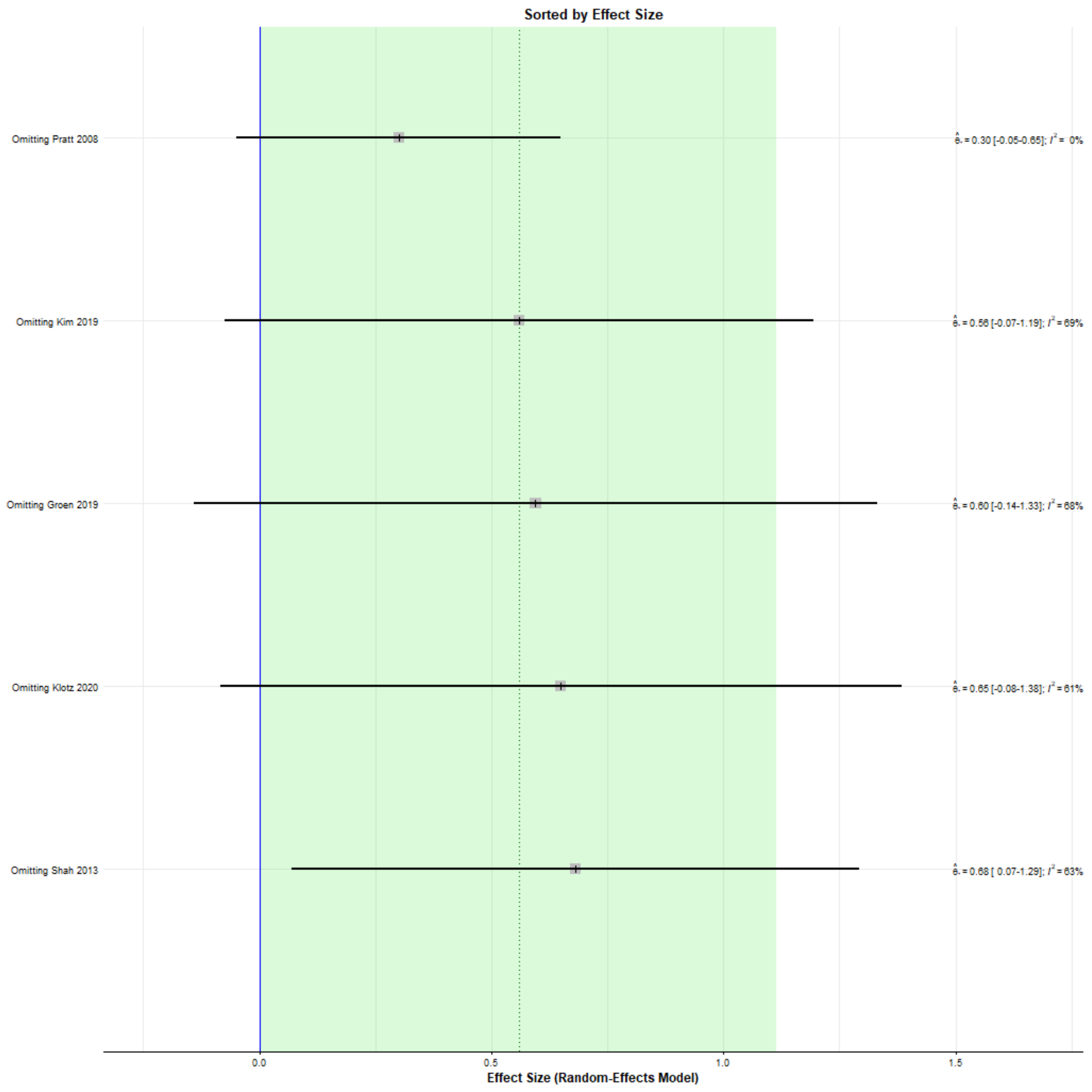

Supplementary Figure S2. Leave-one-out sensitivity analysis plot for selected studies for the pain scores on postoperative day 2 in patient-controlled analgesia versus epidural analgesia.

$\hat{\Theta}$ = mean difference

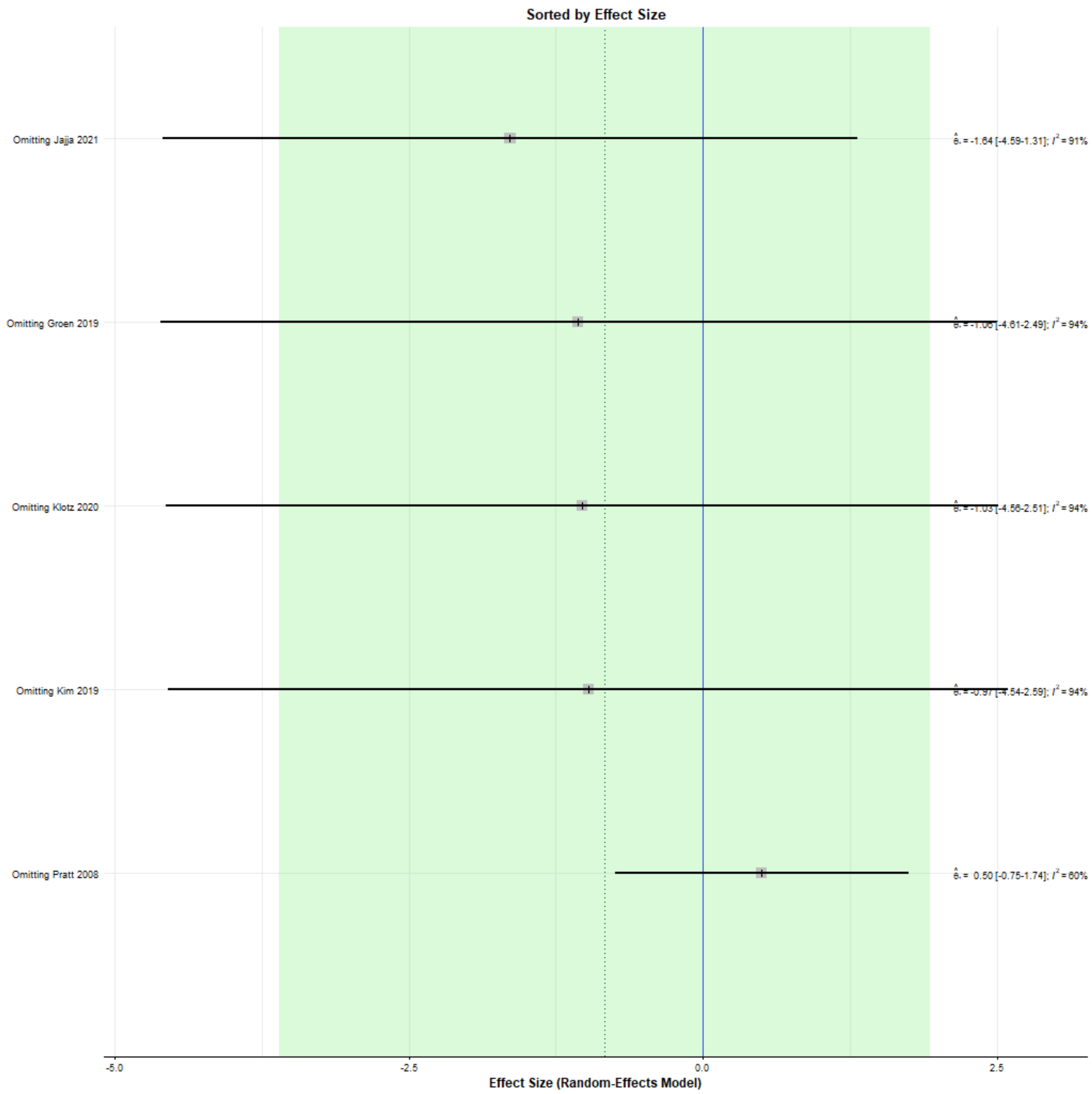

Supplementary Figure S3. Leave-one-out sensitivity analysis plot for selected studies for the length of stay (days) in patient-controlled analgesia versus epidural analgesia.

$\hat{\Theta}$ = mean difference
